# Mycoproteins and Beyond: Biotechnological Advances in Filamentous Fungal Biomass

**DOI:** 10.2174/0113895575399738251007060021

**Published:** 2026-06-08

**Authors:** Parwiz Niazi, Abdul Bari Hejran, Khaidarov Saken, Kakimova Ardak, Indra Jeet Chaudhary, Neha Agarwal, Virendra Kumar Yadav

**Affiliations:** 1 Department of Biology, Faculty of Education, Kandahar University, Kandahar, 3801, Afghanistan;; 2 Department of Biology, Faculty of Education, Helmand University, Lashkar Gah, Helmand No. 3901, Afghanistan;; 3 Department of Biotechnology, Faculty of Biology and Biotechnology, Al-Farabi Kazakh National University, 71 Al-Farabi Avenue, Almaty, 050040, Kazakhstan;; £ Department of Molecular Biology and Medical Genetics, Asfendiyarov Kazakh National Medical University, Zheltoksan Street 37A, Almaty 050004, Kazakhstan;; 4 Department of Environmental Science, Savitribai Phule Pune University, Ganeshkhind Road, Pune, Maharashtra, 411007, India;; 5 Department of Chemistry, Navyug Kanya Mahavidalaya University of Lucknow, Lucknow, India;; 6 Marwadi University Research Center, Department of Microbiology, Faculty of Sciences, Marwadi University, Rajkot, 360003, Gujarat, India

**Keywords:** Alternative protein market, circular bioeconomy, filamentous fungi, immunomodulatory metabolites, mycoprotein, SDG 2

## Abstract

The rapid growth of the global population has increased the demand for animal-based proteins, placing considerable pressure on land, feed resources, and space, ultimately making their production costly and environmentally taxing. Filamentous fungi present a promising alternative, serving as a source of mycoprotein and pharmaceutical products. They are rich in high-quality proteins with favorable amino acid profiles, positioning them as an eco-friendly replacement for conventional animal- and plant-derived proteins. In addition, these fungi produce a wide array of secondary metabolites with antibacterial, anticancer, and immunomodulatory properties, highlighting their value in drug discovery. This review discusses recent progress in fungal biomass production through solid-state and submerged fermentation, along with their nutritional relevance and applications in food technology. Importantly, filamentous fungi can grow on inexpensive agricultural by-products, improving both economic feasibility and environmental sustainability. Their bioactive metabolites also hold strong potential for tackling pressing health issues, establishing filamentous fungi as a versatile biotechnological tool for advancing sustainable food systems and pharmaceutical innovation. The present study contributes to multiple UN Sustainable Development Goals by promoting SDG 2 (zero hunger) through sustainable fungal proteins that enhance food security and nutrition, and SDG 3 (good health and well-being) *via* bioactive metabolites with therapeutic potential. It advances SDG 9 (Industry, Innovation, and Infrastructure) through innovations in fermentation, genetic engineering, and bioprocessing, while supporting SDG 12 (responsible consumption and production) by utilizing agro-industrial waste within a circular bioeconomy. Moreover, SDG 13 (climate action) is addressed, as mycoprotein production significantly reduces greenhouse gas emissions compared to livestock.

## 1 INTRODUCTION

As the global population grows, there is a rising need for nutritious, sustainable, and affordable food sources [1]. The increasing human population predominantly depends on flora and fauna for food. Addressing the growing demand for plant-based products requires the cultivation of additional crops, hence necessitating expanded land use. Likewise, the production of animal-based commodities necessitates substantial quantities of feed, making the process both expensive and inefficient. Research indicates that traditional cattle production significantly harms the environment, human health, and ethical standards, emphasising the urgent necessity for alternatives [2]. Recent studies indicate that the global animal rearing and livestock production sector generates more greenhouse gases than the emissions produced by the worldwide transportation industry, including vehicle exhaust [3]. Increasing concerns are growing over the health hazards of processed meat and the increased cholesterol levels linked to animal-based diets [4]. This highlights the need for alternative food sources that are safer, more economical, and less resource-intensive compared to conventional plant and animal products. Microorganisms, particularly fungi and filamentous fungi, represent a feasible alternative.

Filamentous fungi are well known for the production of various enzymes that offer a substitute for meat in terms of nutritional value and economic feasibility [5]. These filamentous fungi can be either macroscopic or microscopic fungi, depending on their size and how easily the eye can see the fruiting body (such as the button mushroom, *Fusarium* spp*.*) [6]. While some microorganisms, such as filamentous fungi, can be multicellular, single-cell protein (SCP) generally refers to protein obtained from microorganisms like yeast, fungi, algae, and bacteria, which utilize different carbon sources for their synthesis [7]. This microbial protein, called SCP, is produced during fermentation [7-9]. These fungi could increase the nutritional value of the substrates by improving nutrient bioavailability and absorption in the human digestive system. Moreover, these filamentous fungal biomass have several bioactive compounds (BaCs) like antioxidants, minerals, polyunsaturated fatty acids, and fibres, which provide health benefits and lower the risk of various diseases [4]. Several filamentous fungi have been used as food in various forms; for instance, *Fusarium venenatum* is a well-known edible filamentous fungus whose mycoprotein is marketed under the brand name Quorn [10]. Bogale *et al.* (2023) reported that most microorganisms utilised for SCP production possess 40% or greater crude protein content on a dry weight basis [11]. The media and production protocols for fungal cultivation focus on the large-scale synthesis of mycoprotein and edible filamentous fungi for industrial and medical uses [12].

The BaCs produced by these filamentous fungi are essential in drug formulation. Fungi produce secondary metabolites, such as antibiotics, immunosuppressants, and statins, which are essential for pharmaceutical development [13]. The ability of these fungi to produce BaCs and high protein biomass presents new opportunities for drug discovery and advancements in food technology [14]. Filamentous fungi are important for the production of proteins, enzymes, metabolites, organic acids, vitamins, and polyunsaturated fatty acids. Additionally, their biomass is a valuable resource for the food, textile, and other industries [13, 15].

The USFDA has authorized various fungal species, such as *Aspergillus, Neurospora, Rhizopus*, and *Fusarium*, for human consumption. Notably, *Aspergillus* and *Neurospora* have been utilised in Asian cuisines and beverages for centuries [16]. The protein content of filamentous fungi can reach as high as 50%, contingent upon the specific type of fungi and the cultivation conditions employed [17]. The higher protein content of fungal biomass makes it a viable source of protein for human consumption [18]. Despite being well-established processes, solid-state fermentation (SSF) and submerged fermentation (SmF) remain underexplored in their potential to enhance protein-rich food production and medicine discovery.

The present study aligns with several UN Sustainable Development Goals. SDG 2 (Zero Hunger) is supported through the use of filamentous fungi as sustainable protein sources that enhance food security, nutrition, and dietary diversity. SDG 3 (Good Health and Well-being) is addressed by the production of fungal secondary metabolites with antimicrobial, anticancer, and immunomodulatory properties that advance drug discovery and healthcare. SDG 9 (Industry, Innovation, and Infrastructure) is reflected in biotechnological advancements, including fermentation strategies, genetic engineering, and large-scale bioprocessing. SDG 12 (Responsible Consumption and Production) is promoted by valorizing agro-industrial waste within a circular bioeconomy, thereby ensuring sustainable protein production while reducing waste and resource dependency. Finally, SDG 13 (Climate Action) is supported since mycoprotein production offers a much lower greenhouse gas footprint compared to livestock, helping mitigate climate change.

This review emphasises the potential of filamentous fungal biomass for the development of protein-rich foods and valuable bioactive compounds for pharmaceutical applications. Further, authors reviewed the function of these fungi as a cost-effective and sustainable substitute for traditional protein sources. Further emphasis was given on the comparison between submerged and solid-state fermentation techniques regarding their effectiveness in enhancing biomass production and maximising the yield of bioactive compounds. Further authors reviewed the nutritional value and pharmacological benefits of fungal proteins and secondary metabolites, highlighting their significance in enhancing food security and promoting therapeutic applications. Finally, the authors emphasized identifying current challenges in large-scale fungal biomass production and proposed future opportunities for innovation in biotechnology and industrial applications.

## 2 STUDY DESIGN AND METHODOLOGY

The reviewed literature was gathered through a comprehensive search of electronic databases (Scopus, Web of Science, PubMed, ScienceDirect, and Google Scholar) using particular keywords related to filamentous fungi, mycoproteins, protein production, and sustainability. Studies published in English between 2000 and 2024 were considered. The inclusion criteria focused on peer-reviewed articles, book chapters, and reports directly addressing protein production, functional properties, environmental impact, and applications of filamentous fungi. Irrelevant, duplicate, or non-English studies were excluded. The collected works were then screened and thematically analyzed to identify key trends, applications, and research gaps.

## 3 FUNGAL BIOTECHNOLOGY

Fungal biotechnology includes several applications utilizing fungi's unique biochemical and genetic potential across agriculture, medicine, industry, and environmental management. Fungi play pivotal roles in these domains due to their ability to produce a broad range of enzymes, BaCs, and other valuable products [19]. In industries, fungi are mainly utilized for the production of enzymes (amylases, cellulases, and lipases), organic acids (citric acids, kojic acid, *etc*.), vitamins (riboflavin, *etc*.), mycoprotein (single cell protein), and many more products [20]. All these myco-derived products are essential for food processing, textile manufacturing, and biofuel production. Besides this, several fungi are agriculturally very important due to their role as biocontrol agents or biofertilizers.

Fungi act as biocontrol agents used to control plant pathogens and reduce the use and dependency on chemical pesticides. Furthermore, some of the filamentous fungi play a crucial role in the bioremediation of harmful chemicals and pollutants present in the soil and water [21]. Besides these, filamentous fungi are also widely used in the pharmaceutical sector, especially for the manufacturing of antibiotics like penicillin and other compounds.

They also have an important role in sustainable food production; for instance, *Aspergillus* and *Fusarium* are used to produce protein-rich foods such as mycoprotein (*e.g.*, Quorn), offering an eco-friendly alternative to meat [22]. Besides this, some of the filamentous fungi are also used for fermentation, especially for the production of beer, wine, and sake. Recently, some of the fungi’s potential has been exploited for the production of biodegradable polymers and, subsequently, their utilization in preparing packaging materials. In all these sectors, the enhanced yields can be obtained by using genetic engineering of the filamentous fungi. Genetic engineering could be applied for enhanced enzyme production, metabolic pathways, and stress tolerance [23]. Besides this, omics technologies (genomics, transcriptomics, proteomics, and metabolomics) and bioinformatics have also played a valuable role in decoding fungal biology, identifying valuable genes, and designing genetic modifications for tailored biotechnological applications.

From the above information, it could be concluded that fungal biotechnology may play a crucial role in addressing various societal and environmental issues while driving innovation and sustainability in diverse sectors [24].

## 4 NUTRITIONAL VALUE OF FILAMENTOUS FUNGI

Filamentous fungi provide numerous nutritional benefits, which make them a promising resource for enriching diets and addressing global food security challenges. Among them, species, such as *Aspergillus*, *Penicillium*, and *Fusarium* are recognized for their high nutritional value [25]. A significant characteristic of these fungi is their increased protein content, which may surpass that of traditional protein sources, such as meat and legumes [25, 26]. Their mycoproteins provide all essential amino acids required for various physiological functions in the human body [6, 27].

Filamentous fungi are abundant in dietary fibre, which contributes to digestive health and aids in the regulation of blood sugar levels, in addition to their protein content [28]. Besides this, they are also rich in carbohydrates, including polysaccharides, such as chitin and β-glucans, which enhance energy provision and demonstrate prebiotic characteristics that support immunological regulation [29]. Various studies have focused on the immune-enhancing properties of β-glucans. These fungi are significant sources of vitamins, including riboflavin, niacin (B3), and folate (B9), as well as minerals, such as iron, calcium, and zinc, which are crucial for numerous biochemical processes [30].

Filamentous fungi make a lot of BaCs, such as polyphenols and antioxidants, that help lower oxidative stress and inflammation, which in turn reduces the risk of chronic diseases. This occurs alongside the presence of macronutrients and micronutrients [6, 28]. Filamentous fungi, owing to their nutritional density and high bioavailability, present a viable solution for improving dietary diversity and meeting nutritional needs, particularly in populations facing malnutrition or lacking access to traditional protein sources [31]. Their nutritional content, adaptability, and sustainability highlight their promise as a feasible solution to global food security issues. Table **1** presents a summary of the nutritional values associated with different filamentous fungi [32-34].

The table indicates that filamentous fungi hold great importance due to their nutritional benefits, particularly as a rich source of protein, containing approximately 50% protein and 45% essential amino acids. The amino acid bioavailability is (81%-92%), which is notably high. They have higher carbohydrate content (11.70%-60.90%) and dietary fibre (4.32%-41.13%). Besides this, there is moderate lipid (0.84%-3.88%) and ash (3.91%-10.92%) content. They are rich in various essential vitamins (such as riboflavin, B3, and folates) and minerals (K and Cu). Moreover, fungi act as a worthy source of energy, providing between 319.9 and 363.1 kcal per 100 g.

## 5 FILAMENTOUS FUNGI *VS*. OTHER MICROBIAL PROTEINS (ALGAE, YEASTS, BACTERIA)

Filamentous fungi possess several distinctive features that set them apart from other sources of microbial protein, such as algae, yeasts, and bacteria. Their multicellular hyphal growth and adaptability to both SSF and SMF give them a unique position in biotechnology [35, 36]. Filamentous fungi are known for producing and secreting large amounts of proteins, particularly enzymes, supported by advanced secretory pathways and post-translational modifications that enhance protein stability and function [37, 38]. Species, such as *Aspergillus niger* and *Trichoderma reesei,* are well known for their high enzyme yields, especially cellulases and proteases. At the same time, comparative studies show that filamentous fungi can adapt protein expression to specific substrates, improving their efficiency in biomass deconstruction.

A major advantage of these fungi is their ability to grow on simple, low-cost substrates, including agricultural residues and industrial by-products, which makes them more sustainable than many other microorganisms that often rely on nutrient-rich or expensive media [39]. In submerged cultures, the filamentous form of fungi affects mixing and aeration while aiding in product separation and cell reuse. Iobilization strategies further enhance efficiency by simplifying cell recovery and enabling continuous production [38]. Filamentous fungi possess a significant advantage in their ability to perform complex post-translational modifications, including glycosylation, which are often absent in bacterial systems [37]. These organisms serve as optimal hosts for the synthesis of functional proteins. Besides industrial enzyme production, these fungi play a crucial role in biodegradation and mycoremediation due to their capacity to degrade lignocellulosic biomass and other resistant polymers [40, 41]. Proteomic studies of the extracellular secretomes from filamentous fungi have highlighted their potential in various industries, including food, pharmaceuticals, and biofuels [3, 42, 43].

Nutritionally, filamentous fungi provide a complete amino acid profile and favorable fatty acid composition, with protein content reaching about 50% of dry weight, essential amino acids contributing nearly half, and digestibility values of 81–92% [44, 45]. Their proteins possess excellent gelling, emulsifying, and texture-forming properties derived from the mycelial network and cell wall polysaccharides, such as β-glucans and chitin, making them especially useful in meat substitutes like Quorn [12]. Yeasts and bacteria also contain high protein levels but require nucleic acid reduction during processing, whereas algae provide additional benefits, such as omega-3 fatty acids that support cardiovascular health [46].

From a sustainability point of view, filamentous fungi offer strong advantages due to their ability to utilize low-cost waste streams, while algae contribute through their low land-use footprint and capacity to grow in aquatic environments [12, 44-46]. Yeast and bacteria excel in controlled continuous production but generally lack the same flexibility in substrate use. In terms of cultivation, fungi can be grown in both submerged and solid-state systems, with the latter often delivering higher enzyme yields [46, 47]. Algae, however, require light and nutrient inputs, limiting cultivation to specific environments [12], while yeasts and bacteria are relatively easy to grow at scale under controlled conditions [46].

Despite their numerous advantages, fungi also present several challenges. Their biomass requires downstream processing, and potential safety concerns, such as mycotoxins and allergens, must be addressed, although some species are already approved for food use [28]. Scaling up can be complicated by issues with oxygen transfer in SMF or moisture control in solid-state systems, though reactor design improvements and morphological engineering offer solutions [12, 46, 48].

Overall, filamentous fungi combine high protein quality, strong functional properties, and substrate versatility, which gives them a clear edge for applications where texture and functionality are important [6]. Yeasts like *Saccharomyces* and *Yarrowia* are robust and easy to scale, but lack filamentous structure. Bacteria grow rapidly and achieve high productivity but depend on sterile, defined media, with most applications focused on feed or biochemical production. Algae, such as *Spirulina* and *Chlorella,* can accumulate 30-70% protein while also providing valuable lipids and pigments [49]. However, their reliance on light, water, and land makes them less cost-effective unless integrated into waste-based systems. Table **2** provides a major difference between filamentous fungi and other microorganisms as an alternative source of protein [50].

## 6 DIFFERENT MEDIA AND SUBSTRATES FOR THE PRODUCTION OF FUNGAL BIOMASS

The production of filamentous fungal biomass involves the utilization of various media and substrates, where each offers unique advantages and drawbacks [51]. These substrates can be broadly categorized into solid and liquid media, with each type being suitable for different fungal species and production goals (Fig. **1**) [52]. The choice of substrate significantly influences the growth rate, biomass yield, and quality of the fungal product.

### 6.1 Solid Substrates

Solid substrates are often preferred for their ability to mimic natural fungal habitats, providing a rich source of nutrients and structural support for fungal growth.

#### 6.1.1 Agricultural by-products

Solid substrates, such as pearl millet, maize, sweet corn, and various pulses like green gram and chickpea, have been effectively used for the mass production of *Metarhizium anisopliae*. Among these, green gram and wheat bran were found to be particularly effective, yielding high conidial counts and biomass [53]. Likewise, alternative substrates like soybean cake and wheat seed cake, have exhibited high biological efficiency for the cultivation of various mushroom species, which transforms agro-wastes into valuable mycelia [54].

#### 6.1.2 Lignocellulosic Materials

These materials, derived from plants and other plant-based substrates, act as a sustainable substitute for the cultivation of fungi. They have a surplus amount of different nutrients required for the growth of fungi, which makes them a potential material for producing mycological biopolymers [55].

#### 6.1.3 Solid-state Fermentation (SSF)

This method is beneficial for fungi due to their potential to grow on solid particles, which utilize carbon and mineral nutrients through enzymatic degradation. SSF is mainly suitable for producing secondary metabolites and enzymes [56].

### 6.2 Liquid Media

Liquid media are often used for controlled and scalable fungal biomass production, allowing precise manipulation of growth conditions.

#### 6.2.1 Synthetic Media

Sabouraud dextrose medium with yeast extract has been identified as a superior liquid medium for the growth of entomopathogenic fungi, such as *Aseauveria bassiana* and *M. anisopliae*, supporting high biomass and conidial viability [57].

#### 6.2.2 Vinasse-based Media

This medium, derived from sugar-to-ethanol plants, supports efficient fungal growth without additional nutrient supplementation. It proves especially beneficial for filamentous fungi, offering a sustainable and economical approach to biomass production [58].

#### 6.2.3 Custom Formulations

Liquid media can be tailored with particular carbon and nitrogen sources, including sucrose and sodium nitrate, as well as necessary minerals to enhance fungal growth. These formulations facilitate the swift cultivation of filamentous fungi while effectively suppressing bacterial growth [59].

No doubt, both solid and liquid media have their advantages and drawbacks. Still, the selection of the substrate mainly depends on the types of fungal species and the intended application of the biomass. During the utilization of agro-waste, solid substrates are primarily preferred as they are more sustainable and cost-effective [60]. During industrial-scale production, greater control over growth conditions is required, due to which liquid media is preferred. Moreover, the environmental impact and availability of substrates are important factors during the selection of the appropriate medium for fungal biomass production [61, 62].

The biomass of filamentous fungi can be effectively obtained by using different media and substrates. Each of these media has its benefits and drawbacks. The specific requirements of the fungal species and the desired features of the biomass product should guide the choice between solid and liquid media [62]. Table **3** shows different media utilized for growing filamentous fungal biomass along with their application [63-77].

## 7 PRODUCTION TECHNIQUES FOR FILAMENTOUS FUNGAL BIOMASS

Fungal biomass production has become increasingly significant due to its applications in biotechnology, pharmaceuticals, and environmental remediation. The production process involves various strategies to optimize fungal growth, maximize yield, and enhance the functional properties of the biomass (Fig. **2**) [78]. Here, emphasis has been given to the key production techniques, comparing different fermentation methods, the optimization of growth conditions, scale-up processes, and the role of genetic engineering [66].

### 7.1 Submerged *vs*. Solid-state Fermentation

The two primary methods for fungal biomass production are SMF and SSF. In SMF, fungi grow in liquid media, which provides homogeneous conditions for growth and easier control of parameters like temperature, pH, and oxygen supply [79]. It is preferred for producing fungal metabolites like enzymes and antibiotics. However, it has limitations regarding the oxygen transfer rate, which can affect certain fungi, particularly those with filamentous growth [80].

SSF, on the other hand, involves the growth of fungi on solid substrates without free-flowing liquid. This method is more suited for filamentous fungi, mimicking their natural environment, leading to higher yields of fungal biomass. SSF is also economically favorable since agricultural wastes, such as rice bran, wheat bran, and SBG, can be used as substrates [81]. Although SSF has advantages in yield and cost, it presents challenges in maintaining optimal growth conditions and scaling up production [56]. Table **4** exhibits the benefits and drawbacks of SMF and SSF.

### 7.2 Optimization of Growth Conditions

Optimizing growth conditions is critical for enhancing fungal biomass yield. Key factors that influence fungal growth include temperature, pH, aeration, substrate concentration, and nutrient availability. Each species of fungus has specific requirements, so customizing these parameters is essential for optimal biomass production [86]. For example, temperature affects enzyme activity, fungal metabolism, and cell division. Most fungi grow well between 25°C and 30°C, though thermophilic species may thrive at higher temperatures. Similarly, pH levels must be carefully monitored, as fungi typically prefer a slightly acidic environment (pH 4.0 - 6.0). The nutrient composition of the medium, including carbon and nitrogen sources, also plays an essential role in biomass yield, and optimizing the ratio of these nutrients can significantly enhance growth [87]. Table **5** summarizes the optimized growth conditions for different filamentous fungi and their effects.

### 7.3 Scale-up Processes

Scaling up fungal biomass production from laboratory to industrial levels presents technical challenges. The transition from small-scale batch fermentation to large bioreactors requires careful control of growth conditions, nutrient supply, and aeration [94]. In SmF, scale-up typically requires larger reactor volumes, which may lead to challenges, such as poor oxygen transfer and uneven mixing. Therefore, the design of bioreactors, like stirred-tank reactors and air-lift fermenters, plays a crucial role in maintaining proper oxygen supply and uniform nutrient distribution [95]. In SSF, scaling up presents greater challenges due to the unpredictability of the solid substrate and the complexities of regulating moisture content and aeration [96]. Pilot-scale bioreactors are commonly utilized to identify potential limitations and optimize process parameters before transitioning to industrial-scale production. At larger scales, sophisticated monitoring and control systems are employed to maintain favorable growth conditions [97]. Table **6** provides an overview of the scale-up strategies for filamentous fungi, outlining the techniques applied and their corresponding effects [98-105].

### 7.4 Genetic Engineering Approaches

Genetic engineering has opened new possibilities for enhancing the biomass production of filamentous fungi. It is possible to increase the efficiency of nutrient utilization, improve tolerance to environmental stressors, and enhance the production of valuable metabolites through targeted genetic modifications [106]. Techniques such as CRISPR-Cas9 and RNA interference (RNAi) allow for precise editing of fungal genomes [107]. By manipulating genes involved in metabolic pathways, investigators can develop fungal strains with superior growth rates, higher biomass yields, or enhanced production of specific compounds like enzymes or BaCs. Moreover, genetic engineering can help in developing fungi that are capable of utilizing unconventional or cheaper substrates, further decreasing the cost of production [108]. Table **7** summarizes the genetic engineering approaches used for filamentous fungi, detailing the fungi used, the genetic method applied, and the effects observed [109-112].

While genetic engineering has significantly advanced the production capabilities of filamentous fungi, challenges remain, like low transformation efficiency and limited understanding of gene regulation in some species. Moreover, the application of these technologies at the commercial level is often affected by intellectual property issues, particularly with CRISPR technologies. The development of alternative systems, such as Mad7-CRISPR, offers a promising solution to these challenges, expanding the toolbox available for fungal genetic engineering and potentially overcoming some of the existing drawbacks [110].

## 8 FUNCTIONAL PROPERTIES OF FILAMENTOUS FUNGI PROTEINS

Filamentous fungal proteins have functional properties that make them valuable for food and industrial uses by providing versatility, stability, and texture enhancement [26, 113]. Such mycoproteins have superior gelation properties, which allow them to form cohesive and elastic networks in food matrices. Due to this gel-forming ability, these proteins may contribute to the textural attributes of several food products, including meat analogues, plant-based cheeses, and gelled desserts [26]. The gelling properties of mycoprotein can be adjusted by parameters, such as pH, temperature, and protein content, providing versatility in product creation and formulation [114, 115].

The protein obtained from filamentous fungi possesses emulsifying and foaming properties, which are crucial in food preparation and texture improvement by stabilising emulsions and reducing the interfacial tension between oil and water phases [50]. This enhances the stability and longevity of emulsified products, such as dressings, sauces, and spreads. These proteins can generate foams, facilitate aeration, and alter the texture of baked goods, confections, and whipped toppings [116, 117].

Research has demonstrated that mycoproteins possess a strong water-binding ability, which helps preserve moisture and juiciness in food products, while reducing water loss and preventing texture deterioration during storage [9, 118]. The moisture retention capability is advantageous in meat alternatives, as it replicates the succulence and texture of conventional meat products. Additionally, the water-binding properties of these mycoproteins increase the sensory attributes and overall quality of gluten-free baked goods, acting as substitutes for gluten to improve structure and texture [119, 120, 121].

Such mycoprotein is also known for its thermal stability and resistance to proteolysis, which makes it suitable for a vast range of processing conditions, including heat treatment, extrusion, and high-pressure processing [122]. The toughness allows for the incorporation of mycoproteins into various food formulations without compromising their functionality or nutritional integrity [123-125]. Table **8** depicts the functional properties of various filamentous fungal proteins.

From the above table, it could be concluded that filamentous fungi have different functional roles in food production. *Pleurotus* spp. is known for high protein levels, essential amino acids, and health-promoting properties, including anticancer, antibacterial, and immunomodulatory functions, alongside notable water retention and emulsification capabilities. *P. expansum* and *P. limosum* provide antifungal proteins and improve food texture, respectively. *F. venenatum* and *A. oryzae* are known for their high protein content and their ability to regulate amino acids while allowing for continuous production. *M. purpureus* effectively processes waste substrates, and edible mushrooms present a comprehensive essential amino acid profile with high digestibility.

## 9 FILAMENTOUS FUNGI: A SOURCE OF PHARMACEUTICAL PRODUCTS

Some of the BaCs produced by fungi have medical values, which are mainly produced by fermentation. Specific fungi, like *Penicillium* and *Aspergillus,* are selected for their potential to produce valuable compounds [130, 131]. In particular, the process of utilizing fungi for the manufacturing of drugs begins with the careful selection of specific fungal species known for their ability to produce BaCs with medicinal properties. Further, these fungi are then cultivated in controlled conditions, such as large fermentation tanks or bioreactors. Here, these fungi undergo fermentation and naturally synthesize valuable compounds [132]. After cultivation, fungi are harvested, during which the BaCs are extracted from the fungal biomass. Further, the extracted BaCs are purified by filtration and chromatography methods to ensure pharmaceutical-grade purity. The purified BaCs are formulated into various dosage forms suitable for administration, including tablets, capsules, or injections [133]. Throughout the entire production method, stringent quality control measures are followed to ensure the safety, potency, and purity of the final drug product. This process harnesses the natural biochemical potential of fungi to produce drugs that can effectively treat a wide range of diseases [134]. Table **9** shows the list of drugs produced by filamentous fungi along with their applications [135-140].

Filamentous fungal species produce several BaCs with significant therapeutic and pharmaceutical applications. Fungi like *Penicillium chrysogenum* and *Acremonium chrysogenum* are known for their production of important antibiotics like penicillin and cephalosporin, respectively. Other species like *Aspergillus fumigatus* and *Scedosporium* spp*.* produce drugs like Olorofim, which is effective against drug-resistant strains and mold pathogens. Fungi-derived medicines are used for antifungal, antibacterial, anticancer, and immunosuppressive purposes. This exhibits the broad applications of fungi in drug development, mainly in addressing diseases, cancer, and immune system modulation.

## 10 ENVIRONMENTAL IMPACT OF FILAMENTOUS FUNGI

The environmental effects of filamentous fungi depend on where and how they are used. They are important for the ecosystem because they help break down organic matter and help plants grow [141]. They have also become popular as a sustainable source for producing protein-rich foods and drugs, which is beneficial for the environment. They can turn low-cost by-products into foods that are high in nutrients, which is better for the environment than traditional protein sources like meat, which are detrimental to the environment [39, 142]. These fungi can produce a wide range of bioactive compounds, making them valuable in pharmaceutical development. Their dual role as both food and medicine emphasizes their potential in tackling global challenges of food security and health. This section explores the environmental impacts of filamentous fungi across various sectors.

As mentioned, filamentous fungi, such as *A. oryzae,* play an important role in producing protein-rich foods. They can grow on agro-waste and turn it into high-protein biomass. This approach not only helps reduce waste but also provides a sustainable alternative to animal farming, with a much lower environmental footprint [6, 44, 45]. Fungal proteins are highly nutritious, offering a well-balanced amino acid profile and rich sources of essential fatty acids. They are also high in fibre, low in fat, and provide versatile options that can meet a wide range of dietary needs [31, 44]. Growing these fungi supports the idea of a circular economy, as they can thrive on agro-industrial waste, helping cut down overall waste generation while reducing the demand for additional agricultural inputs [44, 143, 144]. Consumer acceptance of fungal-based foods is also growing, with products such as fungal burgers, showing potential in the market, and studies even reporting that some consumers prefer their taste and texture over conventional meat alternatives.

Beyond food applications, filamentous fungi have also emerged as valuable resources in drug development; for instance, these fungi produce BaCs (enzymes, secondary metabolites), which have significant pharmaceutical applications. Their metabolic diversity makes them a promising platform for the discovery and production of novel drugs and therapeutic agents [26, 47]. In addition to pharmaceutical applications, the strong secretory capabilities of filamentous fungi are also utilized in environmental remediation, reinforcing their importance in sustainable biotechnology [47].

Literature has shown that during the production of alternative proteins and drugs from filamentous fungi, a requirement for growth media exists. So, if the growth media is developed from the waste products like agro waste (corn cob, husk from wheat and rice, shells from fruits, fruit peels from industry *etc*), paper and pulp industry waste, dairy waste (whey protein, soy protein) then not only the growth media becomes economical but also the synthesized product from these fungi [145]. Moreover, the utilization of agro-waste for filamentous fungi reduces the solid waste-related problem and may help in preventing various types of pollution in the environment. Otherwise, the burning or landfilling of these solid and liquid waste may further require energy and labor, and may result in economic loss [146].

In addition to the valorization of agro waste, filamentous fungi protein can have a substantially lower greenhouse-gas (GHG) footprint than ruminant meat when normalized by protein, and it is broadly comparable to leading plant proteins [39]. Recent LCAs for mycoprotein/filamentous fungal protein report cradle-to-gate footprints between 0.73 and 6.15 kg CO_2_e per kg of product (system- and site-dependent) [143, 147]. Using a typical retail protein content of ~13.6% for mycoprotein mince, this corresponds to roughly 5–45 kg CO_2_e per kg of protein; a product-specific LCA for Quorn mince implies ≈31 kg CO_2_e per kg of protein (4.23 kg CO_2_e kg⁻¹ product; 136 g protein kg⁻¹) [148]. By comparison, a global meta-analysis finds beef emits a median of ≈50 kg CO_2_e per 100 g protein (*i.e.*, ≈500 kg CO_2_e per kg protein; 10^th^–90^th^ percentile ≈17–105 kg CO_2_e per 100 g) and soy/tofu typically 2–6 kg CO_2_e per 100 g protein (*i.e.*, ≈20–60 kg CO_2_e per kg protein), with variation by system and geography. These data indicate that, under current practice, filamentous fungal protein reduces GHGs by roughly an order of magnitude compared to beef on a protein-equivalent basis and falls within the same order of magnitude as soy, while retaining the circular-bioeconomy benefit of consuming low-value carbohydrate streams. Given methodological sensitivity (functional unit, energy mix, substrate, allocation), reporting LCAs per 100 g protein and per kg product for the specific process used (substrate, aeration, heat integration, grid mix) is recommended to ensure transparent comparison across studies [149, 150].

Filamentous fungi have some negative impact on the environment, for instance, their cultivation for food or drug production requires resources like land, water, and energy, which can contribute to habitat loss, water depletion, and greenhouse gas emissions if not managed sustainably [151]. Moreover, the release of fungal spores or mycotoxins into the environment, particularly in indoor environments, can cause health hazards to humans and animals [152]. Therefore, the utilization of filamentous fungi in the environment can present risks to human health. For instance, exposure to fungal spores, particularly those from mold species, such as *Aspergillus* and *Penicillium*, can lead to allergic reactions, including respiratory issues and skin irritation [151]. Several fungi produce mycotoxins that can contaminate food [10], potentially leading to acute poisoning or chronic health issues among consumers and workers [113].

In conclusion, no doubt that filamentous fungi play crucial roles in the environment; still, careful management and preventive measures are required to minimize the potential risks to human health associated with their presence and environmental activities [153]. Table **10** summarizes the various environmental impacts of filamentous fungi along with their applications [154-158].

Filamentous fungi show several important roles in the environment and industries. Fungi like *Penicillium* spp*.* and *M. circinelloides* are involved in dairy industries and the degradation of food materials. Moreover, some fungi, such as *P. chrysosporium, P. ostreatus, T. versicolor,* and *L. edodes*, are involved in bioremediation, particularly in the breakdown of industrial contaminants, including phenolic compounds. Fungi like *S. apiospermum* and *C. herbarium* affect the environment negatively through pollution and spoilage.

## 11 ECONOMIC VIABILITY AND MARKET POTENTIAL

The economic viability of filamentous fungal biomass production lies in its potential to meet the growing demand for sustainable protein sources while offering cost-effective production methods; with increasing awareness of environmental concerns and the need for more efficient food production systems, filamentous fungal biomass presents a promising solution [159]. Economically, filamentous fungi can be cultivated on various substrates, including agricultural waste and industrial byproducts. This reduces the production costs and offers a sustainable alternative to conventional protein sources [159]. The rapid growth rate of filamentous fungi allows for high yields in a relatively short time, which further enhances their economic viability [160].

The cost analysis of filamentous fungal biomass production involves evaluating factors, such as substrate costs, labor, equipment, energy, and overhead expenses [63]. One key advantage of filamentous fungi cultivation is its ability to utilize low-cost substrates, including agricultural residues, which can significantly lower production costs in comparison to conventional protein sources. Advances in cultivation methods, like SSF and bioreactor systems, have improved efficiency and reduced labor requirements [159, 161]. Maintaining optimal growth conditions and sterilization adds to production costs, but ongoing research and technological advances are steadily reducing these costs, making filamentous fungal biomass production more competitive [162].

Market trends show growing demand for protein-rich fungal biomass due to sustainability, health awareness, and plant-based diets. The high protein content and complete amino acid profile of filamentous fungi meet consumers' desire for nutritious, wholesome food [118]. The versatility of fungal biomass allows for the production of protein-rich items like meat substitutes, powders, snacks, and supplements. Collaboration among researchers, food manufacturers, and biotech firms drives innovation, expanding the market for fungi-based proteins [25]. The market outlook for protein-rich foods made from fungal biomass is optimistic, with strong potential for growth and market penetration in the coming years [163]. The global mushroom (fungi) market was valued at $57.18 billion in 2022 and increased to $62.44 billion in 2023, reflecting an annual growth rate of 9.2% [164]. It was projected to reach $66.53 billion in 2024 and expand further to $90.88 billion by 2027, with a compound annual growth rate (CAGR) of about 9.8% [165]. Growth is driven mainly by rising demand for mushrooms and processed foods, expansion in emerging markets, and a growing vegan population. The Asia-Pacific region was the largest market in 2022 and is expected to remain the fastest-growing. Major countries contributing to this market include China, India, Japan, South Korea, Australia, Brazil, Russia, the UK, and the US [166]. Leading industry players include Scelta Mushrooms, Monaghan Mushrooms, and Green Yard. Filamentous fungal proteins (mycoproteins such as Quorn) currently represent a small single-digit share of the broader alternative-protein market [167]. Recent market analyses place the global mycoprotein category at ~US$0.63–0.80 billion in 2024–2025, whereas the alternative-protein market totals ~US$18.8–21.5 billion in 2025, implying an indicative ~3–4% share for mycoprotein within alternative proteins (and <1% if very expansive “alternative protein” definitions are used) [168]. Fig. (**3**) shows the mycoprotein market size from 2024 to 2034 (USD million) [168].

Due to their modest market share, several obstacles limit the growth of filamentous fungal proteins. One of them is consumer acceptance**,** which is a major challenge, as products must match conventional proteins in taste, texture, and price while gaining familiarity among buyers [26, 169, 170]. Clear communication about production methods and health benefits can help encourage adoption. Regulatory approval and labeling also pose hurdles. However, Fusarium venenatum–based mycoprotein is recognized as non-novel in the EU and GRAS in the U.S. New fungal strains and applications require separate approvals under Novel Food regulations, which can delay commercialization and lead to labeling restrictions [37, 169]. Besides this, safety and allergens remain a major concern, since a small proportion of consumers experience adverse reactions, highlighting the need for transparent labelling and post-market monitoring. In addition, scaling production is capital-intensive, as fermentation and downstream processing facilities demand high investment, limiting cost competitiveness compared to plant-based alternatives [113, 169]. Overcoming these barriers through product optimization, cost reductions, streamlined regulation, and effective risk communication will be key to realizing the economic potential of filamentous fungal proteins alongside mushrooms in the protein sector [171, 172].

The rise in demand for vegan foods has elevated market growth, as mushrooms are popular among consumers following plant-based diets. These diets, excluding meat, dairy, and eggs, have gained popularity due to health benefits, animal welfare, and environmental concerns [173].

## CONCLUSION

Filamentous fungal biomass offers a sustainable and economically viable alternative to conventional protein sources, owing to its high nutritional value and ability to grow on diverse, low-cost substrates. Advanced fermentation methods, such as solid-state and submerged fermentation, effectively enhance biomass yield and protein quality, while genetic engineering provides further opportunities to improve production efficiency. Beyond food applications, filamentous fungi produce diverse secondary metabolites with antimicrobial, anticancer, and immunomodulatory properties, highlighting their significant role in drug discovery and therapeutic development. Additionally, fungal cultivation supports a circular bioeconomy by converting agro-industrial waste into valuable products, thereby reducing environmental impact. Despite current challenges in large-scale production, safety, and consumer acceptance, the multifunctional potential of filamentous fungi positions them as key contributors to global food security and pharmaceutical innovation. Nonetheless, continued research, technological advancements, and interdisciplinary collaboration will be essential to fully realize their role in shaping sustainable nutrition and modern medicine.

## Figures and Tables

**Fig. (1):**
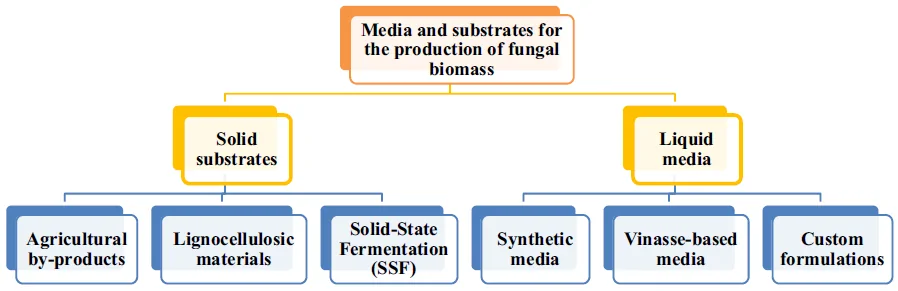
Different media and substrates for the production of filamentous fungal biomass.

**Fig. (2):**
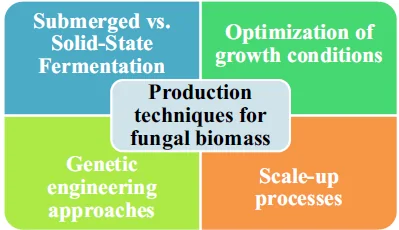
A different method for fungal biomass production.

**Fig. (3):**
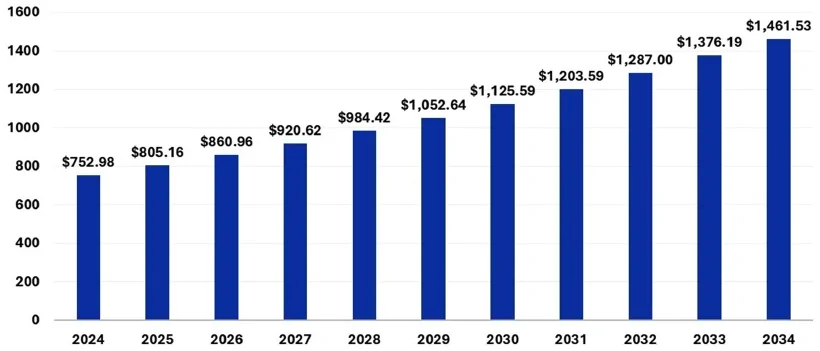
Mycoprotein market size 2024 to 2034 (USD million) [168].

**Table 1:** A summary of nutritional values of different filamentous fungi.

**Nutritional Component**	**Quantity**	**References**
Protein	50% of biomass, with 45% essential amino acids	[17]
Amino acid accessibility	81% – 92%	[31]
Carbohydrates	11.70% – 60.90%	[32]
Lipids	0.84% – 3.88%	[32]
Dietary fibre	4.32% – 41.13%	[32]
Ash content	3.91% – 10.92%	[32]
Vitamins	Rich in riboflavin, B3, and folates	[33]
Minerals	High in K and Cu	[33]
Energy	319.9 to 363.1 Kcal/100 g	[34]

**Table 2:** Comparative major differences between filamentous fungi and other microorganisms as an alternative source of protein.

**Feature**	**Filamentous Fungi**	**Bacteria**	**Yeast**	**Algae**	**References**
Protein yield	High secretion capacity; up to 25 g/L proteins in submerged cultures; glycoproteins 20 - 40 g/L; strong enzyme production	High growth rate; protein yield depends on strain; often requires intracellular extraction (lower secretion efficiency)	Moderate secretion; useful for recombinant proteins, but lower than fungi	Protein 30 - 70% DW; yield depends on light, nutrients, and system	[38, 50]
Secretion efficiency	Excellent – extracellular enzymes/proteins secreted in large amounts; advanced secretory pathways	Generally poor – most proteins remain intracellular; secretion requires genetic engineering	Moderate – some secretion possible, but many proteins are retained intracellularly	Low – proteins are mainly intracellular, requiring cell disruption	-
Post-translational modifications	Extensive, including glycosylation; suitable for functional/recombinant proteins	Limited; most bacteria lack advanced modifications; unsuitable for complex proteins	Good – capable of glycosylation, but less complex than fungi	Limited; PTMs are less studied and less efficient compared with fungi/yeast	-
Cultivation conditions	Grow in diverse, low-cost substrates; flexible morphology (pellets, dispersed mycelia); SSF and SMF possible	Require defined media; efficient in continuous cultures; fast growth under controlled conditions	Easy to scale in SmF; robust growth in industrial bioreactors	Require light, CO₂, and nutrients; cultivation limited to photobioreactors or open ponds	-
Growth rate	Higher than plant/insect/mammalian cells; slower than bacteria but efficient at scale	Very fast; highest among microbes; hours per generation	Fast – slower than bacteria, faster than fungi	Moderate to slow – depends on light/nutrient conditions	-
Functional properties	Excellent – produce enzymes, antibiotics, organic acids; efficient secretion; strong textural and functional properties for food applications	Good for bulk proteins, feed, and biochemicals; limited functionality due to poor secretion and PTMs	Strong for food/beverage fermentation; some functional proteins produced; lacks fibrous structure	Provide omega-3 fatty acids, pigments, antioxidants; additional nutritional benefits beyond protein	-
Industrial use	Enzyme and recombinant protein production, pharmaceuticals, food (*e.g.*, Quorn), biofuels, mycoremediation	Feed proteins (*e.g.*, *Methylococcus*), biochemicals, bioplastics; pharma *via* engineered strains (*e.g.*, *E. coli*)	Brewing, baking, bioethanol, and recombinant protein production	Nutraceuticals (Spirulina, Chlorella), pigments, biofuels, and aquaculture feed	-

**Table 3:** Different media utilized for growing filamentous fungal biomass along with their application.

**Media Used**	**Filamentous Fungi Used**	**Applications**	**References**
Industrial wastewater	*Various filamentous fungi*	Wastewater treatment, biorefinery	[63]
Forest residues and plant wastes	*A. niger, Trichoderma longibrachiatum*	Enzyme production for biofuel	[64]
Powdery form of cheese whey	*Aspergillus oryzae, Rhizopus oryzae, Neurospora intermedia*	Sustainable food production	[65]
Rich and minimal media	*Pleurotus ostreatus, Rhizopus stolonifer*	Biotechnological applications, pellet formation	[66]
Lignocellulosic residues	*Trichoderma lexii (T-94a)*	Composting, waste management	[67]
Diluted thin stillage	*A. oryzae, R. delemar, N. intermedia*	Fish feed ingredient	[17]
Paper substrate	*Alternaria alternata, A. niger, Cladosporium herbarum, Penicillium chrysogenum, Trichoderma atroviride*	Fungal identification and characterization	[68]
Solid substrates (*e.g.*, tree stumps, sawdust)	Various fungi (*e.g.*, mushrooms)	Food production (*e.g.*, mold cheese, tempeh, soy sauce, sake)	[69]
Wheat bran, soybean meal, DDGS, brewer’s spent grain	*A. oryzae*	Protein and polysaccharide-rich biomass for food and feed	[70]
Sugarcane bagasse (SBG)	*Trichoderma koningii, Penicillium* sp*., Rhizomucor* sp*.*	Cellulase enzyme production	[71]
Starchy grains, fibrous substrates, protein-rich substrates	*Fistulina hepatica, Ganoderma lucidum, Pleurotus pulmonarius, Panus lecomtei, Aspergillus terreus*	Nutraceutical enrichment of animal feed	[72]
SBG, soybean flour, CaCO_3_, distilled water	*Rhizopus* sp*.*	Biofoam production	[73]
Various nutrient media with different C and N sources	*G. lucidum, Trametes versicolor*	Biomass synthesis for medicinal purposes	[74]
Vinasse with ammonium sulfate and potassium dihydrogen phosphate	*N. intermedia, A. oryzae*	Protein-rich food production	[75]
Leaves and roots of plants from the Brazilian semiarid	*A. niger*	Hydrolytic enzyme production	[76]
	*Trichoderma harzianum*	β-1,3-glucanase production	[77]

**Table 4:** Advantages and disadvantages of SMF and SSF.

**Parameters**	**SSF**	**SmF**	**References**
**Description**	Filamentous fungi are grown on a solid substrate like agro-waste or waste materials. The biomass produced can be used directly as a protein-rich food source or processed further to extract protein concentrates or isolates	Filamentous fungi are grown in a liquid medium, typically containing a C and N source. The biomass can be directly or further processed to extract protein concentrates or isolates	[80, 82]
**Advantages**	Low cost, simple process, can utilize waste materials, high protein yields	High yields, scalability, ability to control environmental factors, lower risk of contamination	[35, 82-85]
**Disadvantages**	Limited scalability, difficulty in controlling environmental factors, risk of contamination	More complex processes and higher production costs may require additional processing to extract protein	[35, 79]

**Table 5:** Summary of the optimized growth conditions for different filamentous fungi, and their effects.

**Fungi Used**	**Conditions Used**	**Effect**	**References**
*Pleurotus porrigens*	Temperature: 35°C, pH: 5, Nitrogen source: peptone, Metal ions: Mn^2+^ (2 mM)	Highest growth rate (OD 22.8) and enhanced MnP enzyme activity	[88]
*G. lucidum*	Substrate: cotton fibre-rich (75 wt.%), CO_2_: 5 vol.%, Temperature: 26 °C	Dense mycelium with a growth rate of 3.3 mm/day	[86]
*Aspergillus fumigatus*	Temperature: 30°C, pH: 7, N- source: Yeast extract	Highest cellulase activity (3.546 IU/mL) and Fpase activity (0.653 IU/mL)	[89]
*Thermothelomyces thermophiles XJF-9*	Medium: Potato Dextrose Broth (PDB), Incubation: 14 days, Carbon source: dextrose	Maximum growth and production of bioactive secondary metabolites	[90]
*Trichoderma asperellum AU131*	Substrate: Wheat bran + white rice (2:1 w/w), Temperature: 25°C	Highest conidia yield of log10 (8.6)	[91]
*Myrothecium roridum*	Medium: Potato Dextrose Agar, Temperature: 30°C, pH: 5, Photoperiod: 16/8 h	Maximum colony growth (87 mm), Spore production: 504 × 10^6 spores/mL	[92]
*A. terreus*	Medium: PDB, Temperature: 28°C, pH: 5.5, Salinity: 4%	Maximal growth and biomass production	[93]

**Table 6:** Scale-up processes for the mass production of filamentous fungi, including the methods employed and their respective effects.

**Fungi Used**	**Method/Technique Used**	**Effect**	**References**
*A. niger*	Microtiter plate-based cultivation	Enhanced reproducibility and consistent morphology in small-scale tests	[98]
Filamentous fungi (general)	Genetic manipulation and synthetic biology tools	Enhanced heterologous protein production	[99]
Filamentous fungi (general)	Morphological engineering and metabolic regulation	Improved enzyme and chemical production through controlled fungal morphology	[35, 85]
General filamentous fungi	Genetic and metabolic engineering	Enhanced productivity and metabolite formation through strain design	[5]
General filamentous fungi	Morphological engineering	Improved mass transfer and product synthesis by regulating fungal morphology	[35]
General filamentous fungi	Stirred tank reactors with Rushton turbine and hydrofoils	Improved O_2_ and CO_2_ mass transfer, reduced mechanical stress	[100]
General filamentous fungi	Suspended nutritionally solid substrate in an air-lift bioreactor	Increased yield of fungi and metabolites by combining solid-state and submerged culture	[101]
*N. intermedia*, *A. oryzae*	Vinasse-based media cultivation in an airlift bioreactor	Efficient fungal biomass production with limited ethanol production	[102]
*P. ostreatus*	SSF with steam and lime pasteurization	Improved nutritional value and digestibility of lignocellulosic biomass for ruminant feeding	[103]
*Talaromyces albobiverticillius*	Scale-up from shake flask to stirred bioreactor with controlled pH and oxygen levels	Enhanced pigment production and biomass growth; pellet morphology in bioreactor	[104]
Various filamentous fungi	Bioreactor fermentation with optimized aeration, pH, and temperature	Increased production of microbial inoculants and metabolites	[105]

**Table 7:** Summary of various genetic engineering approaches used for filamentous fungi, detailing the fungi used.

**Fungi Candidate**	**Genetic Technique Used**	**Effect**	**References**
*T. reesei*	Sub-genomic RNAi-assisted evolution	Enhanced cellulase production, 7-fold increase in endoglucanase activity	[109]
*Humicola insolens*	Sub-genomic RNAi-assisted evolution	Increased catalase production, 1.9 times higher than the parent strain	[109]
*Aspergillus* spp*.*	CRISPR-Cas9	Gene disruptions, insertions, deletions; improved strain engineering	[110]
*Aspergillus* spp.	Mad7-CRISPR	Efficient gene editing, marker-free gene editing in multiple species	[110]
*Aspergillus* spp*.*	Homologous recombination, RNA techniques	Construction of efficient fungal cell factories, prevention of mycotoxin production	[111]
Filamentous fungi (general)	CRISPR-Cas9	Enhanced protein secretion, potential for fourth-generation biological products	[5]
Filamentous fungi (general)	Synthetic biology	Enhanced production of high-value metabolites, improved metabolic pathways	[112]

**Table 8:** Functional properties of different filamentous fungi proteins.

**Fungi type**	**Properties**	**References**
*Pleurotus* spp*.*	High protein content with essential amino acids	[126]
-	Anticancer, antitumor, antibacterial, and immunomodulatory effects	-
-	High water holding capacity, emulsifying capacity, and foaming capacity	[127]
*Penicillium expansum*	Production of antifungal proteins (AFPs) with potential for controlling fungal diseases	[128]
-	Involvement in cell wall remodeling and carbohydrate mobilization	-
*Penicillium limosum*	High protein and essential amino acid content	[129]
-	Enhances viscosity, gelling properties, and chewiness in meat analogues	-
*Fusarium venenatum*	High protein content and the ability to regulate amino acid composition	[12]
*A. oryzae*	High protein content and potential for continuous production	[12]
*Monascus purpureus*	High protein content and the use of waste substrates for production	[12]
Edible mushrooms	A complete profile of essential amino acids, along with diverse functional activities	[127]
-	Comparable protein digestibility to common food proteins	[31]

**Table 9:** List of drugs obtained from filamentous fungi along with their applications.

**Name of Fungi**	**Name of Drug**	**Effect of the Drug**	**References**
*Acremonium chrysogenum*	Cephalosporin	Antibacterial	[135]
*A. fumigatus*	Fumagillin	Antiparasitic	[136]
*A. fumigatus*	Olorofim	Potent fungicidal activity against azole-resistant strains and other moulds	[137, 138]
*Aspergillus lanosus*	Griseofulvin	Antifungal, for treating fungal infections of the skin, hair, and nails	[139]
*Aspergillus nidulans*	Echinocandin B	Antifungal, used to treat systemic fungal infections	[139]
*A. nidulans*	Sterigmatocystin	Anticancer	[136]
*Aspergillus species*	Camptothecin	Anticancer, used in chemotherapy for cancer treatment	[139]
*A. terreus*	Lovastatin	Cholesterol-lowering	[135]
*Exophiala species*	Olorofim	Limited activity	[137]
*Fusarium species*	Olorofim	Limited activity against certain species, effective against others	[137, 138]
*Fusidium coccineum*	Fusidic acid	Antibiotic, primarily used against staphylococcal infections	[139]
*Glarea lozoyensis*	Pneumocandin B	Antifungal, precursor to echinocandin antifungal drugs	[139]
*Hypocladium inflatum*	Cyclosporine A	An immunosuppressant, used in organ transplantation to prevent rejection	[139]
*Lomentospora prolificans*	Olorofim	Effective against drug-resistant strains	[137]
*Penicillium chrysogenum*	Penicillin	Antibacterial	[136, 140]
*Penicillium notatum*	Carbapenems	Broad-spectrum antibiotics used for severe or high-risk bacterial infections	[139]
*P. notatum*	Cephalosporins	Antibiotics; used to treat bacterial infections	[139]
*P. notatum*	Monobactams	Antibiotic, effective against Gram-negative bacteria	[139]
*Penicillium species*	Griseofulvin	Antifungal, for treating fungal infections of the skin, hair, and nails	[139]
*Phialocephala fortinii*	Podophyllotoxin	Anticancer, used as a precursor for anticancer drugs like etoposide	[139]
*Scedosporium spp.*	Olorofim	Active against various mould pathogens	[137]
*Taxomyces andreanae*	Paclitaxel	Anticancer, used in chemotherapy for various cancers	[139]
*Tolypocladium inflatum*	Cyclosporine A	Immunosuppressant	[135]
*T. atroviride*	Camptothecin	Anticancer, used in chemotherapy for cancer treatment	[139]
*Verruconis gallopava*	Olorofim	Potent activity, especially compared to other antifungals	[137]

**Table 10:** Various environmental impacts of filamentous fungi, along with applications.

**Name of Fungi**	**Impact on the Environment**	**References**
*Aspergillus* spp.	Causes invasive fungal diseases in immunocompromised patients in hospital environments	[154]
*-*	Acts as a plant pathogen and causes diseases like rust and smuts	[155]
*-*	Produces mycotoxins with health risks in humans and animals	[151]
*Penicillium* spp*.*	Used in the dairy industry, but can cause food spoilage and economic loss	[156]
*-*	Produces mycotoxins with health risks in humans and animals	[151]
*Fusarium* spp.	Causes invasive fungal diseases in hospital environments	[154]
*-*	Produces mycotoxins with health risks in humans and animals	[151]
*Mucor circinelloides*	Associated with natural environments with little anthropogenic impact	[156, 157]
*-*	Used in the dairy industry, but it can cause food spoilage	[156, 156]
*Scedosporium apiospermum*	Associated with environments polluted by sewage	[156]
*Phanerochaete chrysosporium,* *P. ostreatus,* *T. versicolor,* *Lentinula edodes*	Used in bioremediation to degrade phenolic compounds in industrial effluents	[158]
*C. herbarum*	Affects radial growth in the dairy industry, impacting food spoilage	[156]
*Geotrichum candidum*	Affects radial growth in dairy, impacting food spoilage	[156]

## LIST OF ABBREVIATIONS

BaCs: Bioactive Compounds
SCP: Single-cell Protein
SmF: Submerged Fermentation
SSF: Solid-state Fermentation
